# A specialized primal-dual interior point method for the plastic truss layout optimization

**DOI:** 10.1007/s10589-018-0028-9

**Published:** 2018-08-20

**Authors:** Alemseged Gebrehiwot Weldeyesus, Jacek Gondzio

**Affiliations:** 1 School of Mathematics and Maxwell Institute for Mathematical Sciences, The University of Edinburgh, Peter Guthrie Tait Road, Edinburgh, EH9 3FD UK; 2 NNASK Research Institute, Kolska 12, 01-045 Warsaw, Poland

**Keywords:** Truss structures, Linear programming, Interior point methods, Iterative methods for linear systems, 90C05, 90C06, 90C51, 74P05, 65F08

## Abstract

We are concerned with solving linear programming problems arising in the plastic truss layout optimization. We follow the ground structure approach with all possible connections between the nodal points. For very dense ground structures, the solutions of such problems converge to the so-called generalized Michell trusses. Clearly, solving the problems for large nodal densities can be computationally prohibitive due to the resulting huge size of the optimization problems. A technique called member adding that has correspondence to column generation is used to produce a sequence of smaller sub-problems that ultimately approximate the original problem. Although these sub-problems are significantly smaller than the full formulation, they still remain large and require computationally efficient solution techniques. In this article, we present a special purpose primal-dual interior point method tuned to such problems. It exploits the algebraic structure of the problems to reduce the normal equations originating from the algorithm to much smaller linear equation systems. Moreover, these systems are solved using iterative methods. Finally, due to high degree of similarity among the sub-problems after preforming few member adding iterations, the method uses a warm-start strategy and achieves convergence within fewer interior point iterations. The efficiency and robustness of the method are demonstrated with several numerical experiments.

## Introduction

Optimization of truss structures goes back to a seminal work of Michell [26] and has grown to a variety of disciplines in structural optimization for which several advanced formulations dealing with practical requirements, theories on existence and uniqueness of solutions, efficient solution methods, and benchmark problems have been proposed, [2–5, 15, 17, 20–23, 28, 29, 35] to mention only a few.

In this paper, we are concerned with solving the topology optimization problems in plastic design by linear programming. These problem formulations are known to ignore kinematic compatibility conditions. However, for single-load case, their equivalence to the (elastic design) minimum compliance problem has been shown, for examples see [1, 3–5, 17, 36]. For multiple-load case, establishing the equivalence is harder, except for some special cases [31]. Nevertheless, it is worth mentioning that the solution of the simplified linear programming formulations provides a reference lower bound plastic design that can be used at early design stages or as an initial truss layout for multilevel optimization problems, for example [16].

The optimization problems are usually formulated by using a ground structure approach [8] in which a set of nodes is distributed in the design domain and all the possible interconnecting bars are generated. The main goal is then to determine the optimal cross-sectional areas of these bars and obtain the lightest structure that is able to sustain a given set of applied load cases. In order to find the ultimate optimal designs that converge to the corresponding exact solution of the so-called generalized Michell trusses [15, 22, 23] or solutions less sensitive to nodal positions, which may actually be dealt with by non-linear geometry optimization of involving smaller size problems [5], we need to use a very large number of nodes [9]. However, this results in a huge number of possible bars and causes that the underlying optimization problems impose additional requirements on the existing solution techniques. The challenges are apparent for a single-load case problems and increase significantly when multiple-load case problems are dealt with. Although attempts have been made to split the multiple-load case problems into certain set of single-load case problems based on the principle of superposition, they have been successful only for special loading conditions [30]. A need for a rigorous treatment of multiple-load case problems still exists.

An adaptive ground structure approach proposed in [10] has been applied in several studies [33]. It is an iterative procedure, closely related to column generation methods for linear programming [14, 24] where the problems are initially solved for a minimal connecting bars and subsequently members are added until the optimal design is obtained. The technique is very attractive because it relies on solving a sequence of smaller problems and avoids the need of solving the full formulation which is prohibitively expensive due to its excessive size. However, after performing the first few member adding iterations, the size of the sub-problems grows and they require extensive computational effort to reach the solution.

The purpose of this article is to address these challenges by developing a special purpose solution technique based on primal-dual interior point method [37] which is well-known to deliver fast convergence in practice and excel on large-scale problems. For more details and a survey of recent developments in the primal-dual interior point method, we refer the reader to [12] and the references therein.

Interior point methods usually reach a solution after a modest number of iterations. However, a single iteration in these methods might be expensive when very large-scale problems are solved. In such cases, an improvement in the efficiency might sometimes be delivered by replacing direct linear algebra techniques with well-suited iterative methods equipped with efficient preconditioners, see [6, 7, 12] and the references therein.

In this paper, we address several aspects of interior point method implementation and demonstrate how to specialize it to single- and multiple-load case plastic truss layout optimization problems and achieve major improvements of its efficiency. In particular, we focus on three important algorithmic features of IPMs which contribute most to the improvement of the overall efficiency of the method.

First of all, we exploit the algebraic structure of structure of the optimization problems when solving normal equation formulation of the reduced Newton systems. We exploit a particular sparsity structure of the LP constraint matrix to perform implicit eliminations and to reduce significantly the size of these linear equation systems in which we determine the search direction for the virtual displacements. To be precise with the size of the linear systems, for problems on *N*-dimensional design domain, with *d* nodes inter-connected by $n(\gg d)$ member bars, and subjected to $n_L$ independent load cases, we solve linear systems with an $m\cdot n_L\times m\cdot n_L$, $m\approx Nd$, coefficient matrix instead of that with an $(m+n)\cdot n_L\times (m+n)\cdot n_L$ matrix in the case of standard normal equations.

Secondly, we employ iterative methods to solve the already reduced linear systems. We use conjugate gradient method [18, 32] with a preconditioner designed to exploit the particular sparsity structure and other mathematical properties of the reduced geometry matrix.

Finally, we take advantage of the similarity of the sequence of problems after some of the first few member adding iterations. In that case, a warm-start strategy [11, 13] is used to define an initial point for the interior point algorithm. This significantly reduces the number of interior point iterations when compared to a cold-start strategy which consists of solving every problem from scratch.

Let us mention at this point that interior point methods have already been applied in the context of truss topology optimization problems. A specialized variant of such method was used in [19]. The linear systems applied to compute search directions were reduced to involve only the displacement variables and one dual variable associated to a volume constraint. The resulting linear systems were dense because no member adding strategy was used. These systems were solved using direct methods of linear algebra. Alternative approaches to truss topology optimization problem include a reformulation as an unconstrained optimization problem using only displacement variables, solved by a gradient descent method [4].

The article is organized in the following manner. In Sect. 2, the overview of a the primal-dual interior point method for a standard linear programming is presented. In Sect. 3, the plastic truss layout optimization, its dual formulation, and the member adding scheme are described. In Sect. 4, the structure-exploiting linear algebra techniques are discussed. In Sect. 5, the iterative method and the applied preconditioner are described. In Sect. 6, the warm-start strategy is explained. The implementation of the method is discussed in Sect. 7 and the numerical results are discussed in Sect. 8. Finally, the conclusions are given in Sect. 9.

## Primal-dual interior point method for linear programming

Consider the standard primal linear problem

1 $$\begin{aligned} \begin{array}{ll} \underset{x}{\text {minimize}} &{} \quad c^Tx \\ \text {subject to} &{} \quad Ax = b \\ &{} \quad x\ge 0, \end{array} \end{aligned}$$

where $A\in \mathbb {R}^{m\times n}$, $x,c\in \mathbb {R}^{n}$, and its dual problem

2 $$\begin{aligned} \begin{array}{ll} \underset{y,s}{\text {maximize}} &{} \quad b^Ty \\ \text {subject to} &{} \quad A^Ty+s = c \\ &{} \quad s\ge 0, \end{array} \end{aligned}$$

where $y,s\in \mathbb {R}^{m}$. In primal-dual interior point methods, we introduce a barrier parameter $\mu >0$ and formulate the perturbed first-order optimality conditions as

3 $$\begin{aligned} \begin{aligned}&Ax = b \\&A^Ty+s =c\\&XSe =\mu e\\&x \ge 0, \ s \ge 0, \end{aligned} \end{aligned}$$

where $X=\text {diag}(x)$, $S=\text {diag}(s)$, and $e=(1,\dots ,1)$ of appropriate size. Then, we solve the system (3) for a sequence of $\mu _k\rightarrow 0$ to find the solution of the primal (1) and dual (2) problems. We apply Newton’s method to the optimality conditions (3) and solve the linear system

4 $$\begin{aligned} \begin{aligned} \begin{bmatrix} 0&A^T&I\\ A&0&0\\ S&0&X \\ \end{bmatrix} \begin{bmatrix} \varDelta x\\ \varDelta y\\ \varDelta s \end{bmatrix} =\begin{bmatrix} \xi _{d}\\ \xi _{p}\\ \xi _c \end{bmatrix}, \end{aligned} \end{aligned}$$

where $\xi _d= c-A^Ty-s$, $\xi _p= b-Ax$, , and $\xi _c= \mu e- XSe$. We follow the Mehrotra’s predictor-corrector method [25] to determine the search directions $(\varDelta x,\varDelta y,\varDelta s)$ in two steps. First, we solve the system (4) with the right hand side $(\xi _d,\xi _p,-XSe)^T$ to find the predictor direction $(\varDelta x_a,\varDelta y_a,\varDelta s_a)$. Then, we determine the maximal primal $\bar{\alpha }_{p}$ and dual $\bar{\alpha }_{d}$ step lengths

5 $$\begin{aligned} \bar{\alpha }_{p}= & {} \max \{\alpha \in (0,1]:x+\alpha \varDelta x_a\ge 0\}\nonumber \\ \bar{\alpha }_{d}= & {} \max \{\alpha \in (0,1]:s+\alpha \varDelta s_a\ge 0\}. \end{aligned}$$

Next, we compute $\mu$ as

6 $$\begin{aligned} \mu =\frac{((x+\bar{\alpha }_{p}\varDelta x_a)^T(s+\bar{\alpha }_{d}\varDelta s_a))^3}{n(x^Ts)^2}, \end{aligned}$$

and solve once again the system (4) with the right hand side $(0,0,\mu e-\varDelta X_a\varDelta s_a)^T$ to find the corrector direction $(\varDelta x_c,\varDelta y_c,\varDelta s_c)$. Finally, we determine the final primal $\alpha _{p}$ and dual $\alpha _{d}$ step lengths as

7 $$\begin{aligned} \alpha _{p}= & {} \tau \max \{\alpha \in (0,1]:x+\alpha ( \varDelta x_a+\varDelta x_c) \ge 0\}\nonumber \\ \alpha _d= & {} \tau \max \{\alpha \in (0,1]:s+\alpha ( \varDelta s_a+\varDelta s_c)\ge 0\}, \end{aligned}$$

where $\tau \in (0,1)$. Then, the new iterate $(x^{+},y^{+},s^{+})$ is

8 $$\begin{aligned} x^{+}= & {} x+\alpha _p ( \varDelta x_a+\varDelta x_c) \nonumber \\ (y^{+},s^{+})= & {} (y+\alpha _d\ ( \varDelta y_a+\varDelta y_c) ,s+\alpha _d( \varDelta s_a+\varDelta s_c) ). \end{aligned}$$

When solving the system (4) in most primal-dual interior point algorithms, the unknowns $\varDelta s$ and $\varDelta x$ are eliminated first and a smaller system called the normal equations is solved.

9 $$\begin{aligned} AXS^{-1}A^T\varDelta y = \xi , \end{aligned}$$

where $\xi$ is the appropriate right hand.

We assume any general solver that uses interior point method would solve the normal equations (9) or other larger systems and perform backward substitution to determine the other directions. Particularly, in case of (9), $\varDelta x$ and $\varDelta s$.

However, for the plastic layout optimization of trusses covered in this article, we can further exploit their algebraic structure and find a much smaller system than (9) which can be efficiently solved. This is described in Sect. 4.

## The plastic truss layout optimization problem

The plastic truss layout optimization problem is formulated following the ground structure approach in which a finite set of nodes, say *d*, are (uniformly) distributed in the design domain. The nodes are then connected by all possible potentials bars $n \gg d$. If the overlapping bars are included, then $n=d(d-1)/2$. We define an optimization problem in which the design variables are the cross-sectional areas $a_i$, $i=1,\ldots ,n$ of the member bars.

Let $m(\approx Nd$, *N* is the dimension of the design domain) be the number of the non-fixed degrees of freedom, $f_\ell \in \mathbb {R}^m,\ell \in \{1,\dots ,n_L\}$ be a set of external forces applied to the structure, and $q^{+}_\ell$, $q^{-}_\ell \in \mathbb {R}_+^n$ be the associated tensile and compressive forces of the bars, respectively.

Then, the multiple-load least-weight truss topology optimization in plastic design can be formulated as

10 $$\begin{aligned} \begin{array}{lll} \underset{a,q_\ell }{\text {minimize}} &{} \quad l^Ta &{} \\ \text {subject to} &{} \quad Bq^{+}_\ell -Bq^{-}_\ell = f_\ell , &{} \quad \ell =1,\ldots ,n_L \\ &{} \quad a\ge \frac{1}{\sigma ^{+}}q^{+}_\ell + \frac{1}{\sigma ^{-}}q^{-}_\ell , &{} \quad \ell =1,\ldots ,n_L \\ &{} \quad a\ge 0 &{} \\ &{} \quad q^{+}_\ell \ge 0, q_\ell ^{-}\ge 0, &{} \quad \ell =1,\ldots ,n_L, \\ \end{array} \end{aligned}$$

where $l\in \mathbb {R}^n$ is a vector of bar lengths, and $\sigma ^{-}>0$ and $\sigma ^{+}>0$ are the material’s yield stresses in compression and tension, respectively. Problem (10) is a linear program. After introducing primal slack variables $x_\ell \in \mathbb {R}_{+}^n,\ell \in \{1,\dots ,n_L\}$ to the inequality constraints and transforming them to equality constraints, i.e., to

$$\begin{aligned} -a+\frac{1}{\sigma ^{+}}q^{+}_\ell + \frac{1}{\sigma ^{-}}q^{-}_\ell +x_\ell =0,\; \quad \ell =1,\ldots ,n_L, \end{aligned}$$

we derive the dual problem associated with (10) given by

11 $$\begin{aligned} \begin{array}{lll} \underset{u_\ell ,y_\ell ,s_a,s_{q_\ell },s_{x_\ell }}{\text {maximize}} &{} \quad \sum f_\ell ^Tu_\ell &{} \\ \text {subject to} &{} \quad -\sum y_\ell +s_a =l &{}\\ &{} \quad B^T u_\ell +\frac{1}{\sigma ^{+}}y_\ell +s_{q^{+}_\ell }=0,&{}\quad \ell =1,\ldots ,n_L\\ &{} \quad -B^T u_\ell +\frac{1}{\sigma ^{-}}y_\ell +s_{q^{-}_\ell }=0, &{} \quad \ell =1,\ldots ,n_L\\ &{} \quad y_\ell +s_{x_\ell }=0,&{} \quad \ell =1,\cdots ,n_L \\ &{} \quad s_a\ge 0 &{} \\ &{} \quad s_{q^{+}_\ell }\ge 0, s_{q_\ell ^{-}}\ge 0,s_{x_\ell } \ge 0, &{} \quad \ell =1,\ldots ,n_L, \\ \end{array} \end{aligned}$$

where $u_\ell \in \mathbb {R}^m$ denotes the virtual nodal displacement, $s_{q^{+}_\ell },s_{q^{-}_\ell },y_\ell \in \mathbb {R}^n, \ell \in \{1,\dots ,n_L\}$, and $s_a\in \mathbb {R}^n$.

### The member adding

**Fig. 1:**
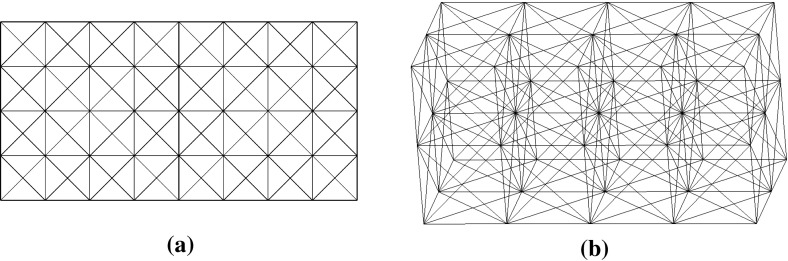
Initial designs. **a** Two-dimensional problems **b** three-dimensional problems

We follow the member adding strategy proposed in [10]. It is an iterative process that starts with a structure constituting a minimum connectivity, see Fig. 1 for example. Let $n_0$ be the number of bars in the initial structure. Let $K_0 \subset \{1,\dots ,n\}$ be the set of indices of the bars for which the optimization problems (10) and (11) are currently solved. Next, we compute the dual violations and generate the set *K* defined by

12 $$\begin{aligned} K=\left\{ j\in \{1,\ldots ,n\}\backslash K_0|\frac{1}{l_j}\sum _{\ell =1}^{n_L}\left( \sigma ^{-}\varepsilon ^{-}_{\ell _j}+ \sigma ^{+}\varepsilon ^{+}_{\ell _j}\right) \ge 1+ \beta \right\} , \end{aligned}$$

where the virtual strains are $\varepsilon ^{+}_{\ell _j} = \max \{(B^Tu_\ell ^{*})_j ,0\}$ and $\varepsilon ^{-}_{\ell _j} = \max \{-(B^Tu_\ell ^{*})_j, 0\}$ with $u_\ell ^{*}$ denoting the optimal virtual nodal displacement and $\beta >0$ some allowed tolerance. Then, the bars with indices in *K* are identified, filtered, and finally added in new problem instance. The member adding process stops when $K=\emptyset$.

There are several heuristics approaches to filter and determine how many of the bars with indices in *K* should be added when formulating the new problem instances. Here, we present three.

1. Include all members in *K*.
2. Sort the members in *K* and include the largest $\min \{\alpha n_0,|K|\}$ members, where $\alpha \le 1$. For example, $\alpha =0.1$ implies at most $10\%$ of the initial number of bars. This is one of the techniques used in [10].
3. Include those in $\{j\in K|l_j\le L_k\}$, where $L_k$ is a limit on the lengths of the bars to be added at the *k*th member adding iteration. Its value increases at every member adding iteration, and reaches the maximum possible length. Particularly, for the numerical experiments in this paper, we use a simple heuristic rule $L_k=2^kr$, where *r* is the diagonal distance between two adjacent nodes. This is motivated by the scheme used in [33].

## Exploiting the algebraic structures

In this section, we describe how the utilize the structure of the least-weight truss layout problems. The primal and dual least-weight truss layout problems (10) and (11) are equivalent to the standard primal-dual linear programming problems (1)-(2) with

13 $$\begin{aligned} x= & {} \left( a, q^{+}_1,\ldots ,q^{+}_{n_L},q^{-}_1,\ldots ,q^{-}_{n_L}, x_1,\ldots ,x_{n_L}\right) \nonumber \\ y= & {} \left( u_1,\ldots ,u_{n_L},y_1,\ldots ,y_{n_L}\right) \nonumber \\ s= & {} \left( s_a, s_{q^{+}_1},\ldots ,s_{q^{+}_{n_L}},s_{q^{-}_1}, \ldots ,s_{q^{-}_{n_L}}, s_{x_1},\ldots ,s_{x_{n_L}}\right) , \end{aligned}$$

and

14 $$\begin{aligned} A= & {} \begin{bmatrix}0&B&\cdots&0&-B&\cdots&0&0&\cdots&0\\ \vdots&\vdots&\ddots&\vdots&\vdots&\ddots&\vdots&\vdots&\ddots&\vdots \\ 0&0&\cdots&B&0&\cdots&-B&0&\cdots&0\\ -I&\frac{1}{\sigma ^{+}}I&\cdots&0&\frac{1}{\sigma ^{-}}I&\cdots&0&I&\cdots&0\\ \vdots&\vdots&\ddots&\vdots&\vdots&\ddots&\vdots&\vdots&\ddots&\vdots \\ -I&0&\cdots&\frac{1}{\sigma ^{+}}I&0&\cdots&\frac{1}{\sigma ^{-}}I&0&\cdots&I \end{bmatrix}\nonumber \\= & {} \begin{bmatrix}0&\quad \tilde{B}&\quad -\tilde{B}&\quad 0\\ -\tilde{I}_v&\quad \frac{1}{\sigma ^{+}}\tilde{I}&\quad \frac{1}{\sigma ^{-}}\tilde{I}&\quad \tilde{I} \end{bmatrix}, \end{aligned}$$

where (borrowing Matlab notation) $\tilde{B}=\text {blkdiag}(B,\ldots ,B)$, $\tilde{I}=\text {blkdiag}(I,\ldots ,I)$, and $\tilde{I}_v=(I,\ldots ,I)^T$. Consequently, the coefficient matrix of the normal equations (9) is

15 $$\begin{aligned} AXS^{-1}A^T=\begin{bmatrix}\tilde{B}\tilde{D}_{11}\,\tilde{B}^T&\quad \tilde{B}\tilde{D}_{12}\\&\\ \tilde{D}^T_{12}\,\tilde{B}^T&\tilde{D}_{22} \end{bmatrix}, \end{aligned}$$

where

16 $$\begin{aligned} \tilde{D}_{11}= & {} \tilde{Q}^{+}\tilde{S}^{-1}_{q^{+}} +\tilde{Q}^{-}\tilde{S}^{-1}_{q^{-}}\nonumber \\ \tilde{D}_{12}= & {} \frac{1}{\sigma ^{+}}\tilde{Q}^{+} \tilde{S}^{-1}_{q^{+}} -\frac{1}{\sigma ^{-}}\tilde{Q}^{-} \tilde{S}^{-1}_{q^{-}}\nonumber \\ \tilde{D}_{22}= & {} \tilde{I}_vA_aS^{-1}_a\tilde{I}^T_v +\frac{1}{(\sigma ^{+})^2}\tilde{Q}^{+}\tilde{S}^{-1}_{q^{+}} +\frac{1}{(\sigma ^{-})^2}\tilde{Q}^{-}\tilde{S}^{-1}_{q^{-}} +\tilde{X}\tilde{S}^{-1}_x \end{aligned}$$

and $A_a=\text {diag}(a)$, $\tilde{Q}^{+}=\text {blkdiag}(Q_1^{+}, \ldots , Q_{n_L}^{+})$ with $Q^{+}_\ell =\text {diag}(q^{+}_\ell )$, $\tilde{Q}^{-}=\text {blkdiag}(Q_1^{-}, \ldots , Q_{n_L}^{-})$ with $Q^{-}_\ell =\text {diag}(q^{-}_\ell )$, $\tilde{X}=\text {blkdiag}(X_1,\ldots ,X_{n_L})$ with $X_\ell =\text {diag}(x_\ell )$, $S_a=\text {diag}(s_a)$, $\tilde{S}_{q_{+}}=\text {blkdiag}(S_{q_1^{+}},\ldots , S_{q_{n_L}^{+}})$ with $S_{q_\ell ^{+}}=\text {diag}(s_{q^{+}_\ell })$, $\tilde{S}_{q_{-}}=\text {blkdiag}(S_{q_1^{-}},\ldots , S_{q_{n_L}^{-}})$ with $S_{q_\ell ^{-}}=\text {diag}(s_{q^{-}_\ell })$, and $\tilde{S}_{x}=\text {blkdiag}(S_{x_1},\ldots ,S_{x_{n_L}} )$ with $S_{x_\ell }=\text {diag}(s_{x_\ell })$.

The matrix in (15) has dimension $(m+n)\cdot n_L\times (m+n)\cdot n_L$ of which $\tilde{D}_{22}$ is an $n \cdot n_L\times n \cdot n_L$ matrix and recall that $n\gg m$. When the problems are solved with general solvers that use the primal-dual interior point method, the Newton system in (4) is at most reduced to the normal equations with the coefficient matrix in (15).

**Fig. 2:**
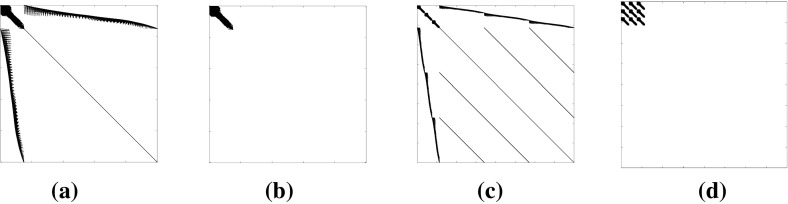
**a** and **c** show the sparsity structure of (15), and **b** and **d** show the sparsity structure of (17). **a** Single-load case, **b** single-load case, **c** three-loads case, **d** three-loads case

**Fig. 3:**
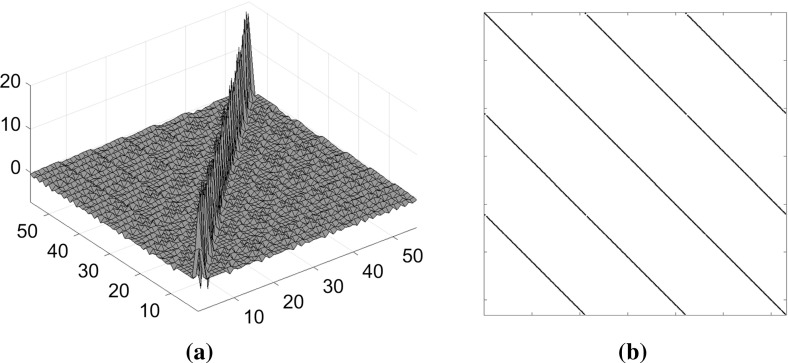
**a** The matrix $BB^T$ for a two dimensional problem. $m=56$, $\max _{i}(BB^T)_{i,i}=20.1058$, $\max _{i}|(BB^T)_{i,i+1}|=6.7788$, and $||BB^T||_F= 108.8457$. **b** The sparsity structure of the matrix $\tilde{D}$ in (17) for three-loads case problem

In this article, we further utilize the structure of the matrix $\tilde{D}_{22}$ which is a diagonal matrix for single-load case problems and built of blocks of diagonal matrices for multiple-load case problems. Example of such structure for a three-load case is displayed in Fig. 3b. In either case, it can be explicitly inverted at almost no cost. Then, instead of solving the normal equations with the larger coefficient matrix (15) which displays structures as in Fig. 2a and c, we solve a much smaller system

17 $$\begin{aligned} \tilde{B}\tilde{D}\tilde{B}^T\varDelta u =\xi _u, \end{aligned}$$

where

18 $$\begin{aligned} \tilde{D}=\tilde{D}_{11}-\tilde{D}_{12}\tilde{D}^{-1}_{22} \tilde{D}^T_{12} \end{aligned}$$

and $\xi _u$ the resulting appropriate right hand side. The coefficient matrix $\tilde{B}\tilde{D}\tilde{B}^T$ has dimension $m \cdot n_L\times m \cdot n_L$ and its corresponding sparsity structures are shown in Fig. 2b and d. Note that for the single-load case problems, the reduction does not even affect the sparsity structure of the block (1, 1) of (15).

### Remark 1

The matrix $\tilde{B}\tilde{D}\tilde{B}^T$ has always a dimension $m\cdot n_L\times m\cdot n_L$. However, its sparsity depends on the member adding iterations.

### Remark 2

Algebraic structure was exploited in interior point method for a nonlinear programming formulation of the minimum compliance problem for truss design developed in [19]. The linear systems in this method were reduced to the ones which involved only the displacements and one extra dual variable corresponding to the volume constraint. The reduced systems in [19] solved using direct methods were completely dense, and no member adding strategy was used.

## Iterative methods for linear systems

Applying direct methods of linear algebra to (17) is challenging due to the size and density of the matrix involved especially for the three-dimensional problems, see Sect. 8.3 and Table 6. Hence, we use the preconditioned conjugate gradient method, that is, we solve the system

19 $$\begin{aligned} M^{-1}\tilde{B}\tilde{D}\tilde{B}^T\varDelta u =M^{-1}\xi _u, \end{aligned}$$

where *M* is a suitable preconditioner. In this section, we propose a preconditioner that well approximates the matrix $\tilde{B}\tilde{D}\tilde{B}^T$ in the sense of Frobenius norm and has the sparsity pattern determined from the detailed features of $\tilde{B}$ and $\tilde{D}$. These are described below for two-dimensional problems. Similar steps can be followed to extend the analysis to three-dimensional problems, see also Remark 3.

We start with analyzing the entries of the matrix $B\in \mathbb {R}^{m\times n}$. Since, theses are direction cosines, we have $|B_{ij}|\le 1, \forall (i,j)$. The number of non-zero entries in each row cannot exceed *m* / 2 which implies $(BB^T)_{ii}\le m/2, \forall i$. Note that, *m* / 2 is the number of nodes in the structure before removing any fixed degrees of freedom. Assembling *B* in the natural way, the sub-diagonal elements of $BB^T$ are the sum of products of sines and cosines of angles which implies $|(BB^T)_{i,i+1}|\le m/4\wedge |(BB^T)_{i-1,1}|\le m/4$. Otherwise, $|(BB^T)_{i,j}|\le 1$. Therefore, the Frobenius norm of $BB^T$ is dominated by the elements on its three diagonals, that is, the entries with indices in the set *T* defined by

20 $$\begin{aligned} T=\left\{ (i,j)\in \mathcal {Z}^m_{++}\times \mathcal {Z}^m_{++}||i-j|\le 1\right\} . \end{aligned}$$

See also Fig. 3a. Moreover, we derive the following bound

21 $$\begin{aligned} ||BB^T||^2_F\le m\Big (\frac{m}{2}\Big )^2+(2m-2)\Big (\frac{m}{4}\Big )^2+(m^2-(3m-2)), \end{aligned}$$

where the first and second terms in the right hand side are contributions from the entries with indices in *T* and the last term accounts for the remaining off-diagonal elements.

Recall that $\tilde{B}=\text {blkdiag}(B,\ldots ,B)$ and $\tilde{D}$ has the sparsity structure displayed in Fig. 3b. Then, the matrix $\tilde{B}\tilde{D}\tilde{B}^T$ has the structure

22 $$\begin{aligned} \tilde{B}\tilde{D}\tilde{B}^T= \begin{bmatrix} B\tilde{D}_{1,1}B^T&\cdots&B\tilde{D}_{1,n_L}B^T\\ \vdots&\ddots&\vdots \\ B\tilde{D}_{n_L,1}B^T&\cdots&B\tilde{D}_{n_L,n_L} B^T \end{bmatrix} \end{aligned}$$

where the block matrices $\tilde{D}_{k,l}$, $k,l\in \{1,\ldots ,n_L\}$ are diagonal. Define the sets

23 $$\begin{aligned} \mathcal {B}=\cup _{k=1}^{n_L}\left\{ i| (\tilde{D}_{k,k})_{ii} \ge \delta \right\} \quad \text{ and } \quad \mathcal {N}=\{1,\ldots ,n\}\backslash \mathcal {B}, \end{aligned}$$

and consequently consider the partition $B=[B_\mathcal {B},B_\mathcal {N}]$ that gives $\tilde{B}=[\tilde{B}_\mathcal {B},\tilde{B}_\mathcal {N}]$ and $\tilde{D}=[\tilde{D}_\mathcal {B},\tilde{D}_\mathcal {N}]$. Then

24 $$\begin{aligned} \tilde{B}\tilde{D}\tilde{B}^T=\tilde{B}_\mathcal {B} \tilde{D}_\mathcal {B}\tilde{B}^T_\mathcal {B} +\tilde{B}_\mathcal {N}\tilde{D}_\mathcal {N}\tilde{B}^T_\mathcal {N}. \end{aligned}$$

Let $n_i$ be the maximum number of non-zero entries in row *i* of $B_\mathcal {N}$. Then $n_i\le m/2, \forall i$. Moreover, $|(B_\mathcal {N}B_\mathcal {N}^T)_{i,i}|\le n_i$, $|(B_\mathcal {N}B_\mathcal {N}^T)_{i,i-1}|\le n_i/2$ and $|(B_\mathcal {N}B_\mathcal {N}^T)_{i,i+1}|\le n_i/2$. Then, the error in the normal equations after dropping the contribution of the $D_{ii},i\in \mathcal {N}$ can be estimated as

25 $$\begin{aligned} ||\tilde{B}\tilde{D}\tilde{B}^T-\tilde{B}_\mathcal {B} \tilde{D}_\mathcal {B}\tilde{B}^T_\mathcal {B}||^2_F= & {} ||\tilde{B}_\mathcal {N}\tilde{D}_\mathcal {N} \tilde{B}^T_\mathcal {N}||^2_F\nonumber \\\le & {} \delta ^2n^4_{L}||B_\mathcal {N}B^T_\mathcal {N}||^2_F\nonumber \\\le & {} \delta ^2n^4_{L}\Big (\sum _i^{m}n^2_i+\sum ^{2m-2}_i \Big (\frac{n_i}{2}\Big )^2+\varDelta \Big ) \end{aligned}$$

where $\varDelta \le (m^2-(3m-2))$ is the less significant contribution from the non-tridiagonal elements.

We propose the preconditioner *M* defined by

26 $$\begin{aligned} (M_{k,l})_{ij}= {\left\{ \begin{array}{ll} (B\tilde{D}_{k,l}B^T)_{ij},&{} \text {if }\quad (i,j)\in T\\ (B_\mathcal {B}\tilde{D}_{\mathcal {B}{k,l}}B_\mathcal {B}^T)_{ij}, &{} \text {otherwise}, \end{array}\right. } \end{aligned}$$

for $k,l\in \{1,\ldots ,n_L\}$. In this case, we have

27 $$\begin{aligned} ||\tilde{B}\tilde{D}\tilde{B}^T-M||_F \le \delta n^2_L \sqrt{\varDelta }. \end{aligned}$$

### Remark 3

For three dimensional problems, the set of indces *T* in (20) is extended to

28 $$\begin{aligned} T=\left\{ (i,j)\in \mathcal {Z}^m_{++}\times \mathcal {Z}^m_{++}||i-j|\le 2\right\} . \end{aligned}$$

### Remark 4

For some of the first few interior point iterations, we use a simpler preconditioner

29 $$\begin{aligned} (M_{k,l})_{ij}= {\left\{ \begin{array}{ll} (B\tilde{D}_{k,l}B^T)_{ij},&{}\quad \text {if }(i,j)\in T\\ 0, &{}\quad \text {otherwise}, \end{array}\right. } \end{aligned}$$

for $k,l\in \{1,\ldots ,n_L\}$ unless the the warm-start strategy described in Sect. 6 is activated.

In Sect. 7, we discuss in more detail the practical effects of using the preconditioners, their implementation, and the spectral properties of linear systems solved.

## Warm-start strategy

Here, we describe the warm-start strategy for truss layout optimization problems. At every member adding iteration described in Sect. 3.1, we generate the set *K* in (12) to identify and add the new members. In that case, the size of the problem grows and the new variables are appended to the problem

30 $$\begin{aligned} (a,q_\ell ^{+},q_\ell ^{-},x_\ell )\rightarrow & {} (a,\bar{a},q_\ell ^{+},\bar{q}_\ell ^{+},q_\ell ^{-}, \bar{q}_\ell ^{-},x_\ell ,\bar{x}_\ell )\nonumber \\ (u,y_\ell )\rightarrow & {} (u,y_\ell ,\bar{y}_\ell )\nonumber \\ \left( s_a,s_{q_\ell ^+},s_{q_\ell ^-},s_{x_\ell }\right)\rightarrow & {} \left( s_a,\bar{s}_a,s_{q_\ell ^+},\bar{s}_{q_{\ell }^+}, s_{q_{\ell }^-},\bar{s}_{q_{\ell }^-},s_{x_\ell }, \bar{s}_{x_\ell }\right) , \end{aligned}$$

where all the variables with the super-bar are vectors in $\mathbb {R}^k$, $k=|K|$, and $\ell \in \{1,\ldots ,n_L\}$.

### Computing a warm-start point

The starting point for the part of the variables in the right hand side of (30) that correspond to the old ones, i.e., those without super-bar, is the solution that is saved while solving the preceding problem with a loose relative optimality tolerance that depends on the level of similarity between the problems, see Sect. 7. This choice of loose tolerances is to avoid points located close to the boundary of the feasible region which could adversely affect the behaviour of interior point methods [11]. Below we propose the initial point for the newly added variables.

We set $\bar{y}$ as

31 $$\begin{aligned} (\bar{y}_\ell )_j =-\sigma _{max}|(\bar{B}^Tu_\ell )_j|-\mu _0^{\frac{1}{2}},\;\forall j\in K, \end{aligned}$$

where $\sigma _{max} =\max \{\sigma ^{-},\sigma ^{+}\}$ and $\mu _0$ is the value of the barrier parameter at the time when the solution was saved. Next, we define the new dual slack variables as

32 $$\begin{aligned} (\bar{s}_a)_j= & {} \max \left\{ \left| \bar{l}_j +\sum _\ell (\bar{y_\ell })_j\right| ,\mu _0^{\frac{1}{2}}\right\} ,\;\forall j\in K\nonumber \\ \bar{s}_{q_\ell ^+}= & {} -\bar{B}^Tu_\ell -\frac{1}{\sigma ^{+}} \bar{y}_\ell \ge \frac{\mu _0^\frac{1}{2}}{\sigma ^{+}},\; \nonumber \\ \bar{s}_{q_\ell ^-}= & {} \bar{B}^Tu_\ell -\frac{1}{\sigma ^{-}}\bar{y}_\ell \ge \frac{\mu _0^\frac{1}{2}}{\sigma ^{-}} ,\;\bar{s}_{x_\ell } =- \bar{y}_\ell ,\quad \forall \ell \in \{1,\ldots ,n_L\}. \end{aligned}$$

Moreover, the new primal variables are set as

33 $$\begin{aligned} (\bar{q}_\ell ^{+})_j= & {} (\bar{q}_\ell ^{-})_j= 0.1\mu _0^{\frac{1}{2}},\;\forall j\in K,\;\forall \ell \in \{1,\ldots ,n_L\}\nonumber \\ \bar{a}= & {} \mu _0\bar{S}^{-1}_a e\nonumber \\ \bar{x}_\ell= & {} \bar{a},\;\forall \ell \in \{1,\ldots ,n_L\}. \end{aligned}$$

Finally, we derive the bounds on the violations of primal and dual infeasibility and complementarity constraints for these newly introduced variables.

#### Primal infeasibility

The primal infeasibilties $\xi _{p_\ell }=(\xi _{p_{1,\ell }}, \xi _{p_{2,\ell }})$, $\ell \in \{1,\ldots ,n_L\}$ are

34 $$\begin{aligned} \left\| \xi _{p_{1,\ell }}\right\| _\infty= & {} ||f_\ell -Bq_\ell ^{+}-\bar{B} \bar{q}_\ell ^{+}+Bq_\ell ^{-}+\bar{B}\bar{q}_\ell ^{-}||_\infty \nonumber \\= & {} ||f_\ell -Bq_\ell ^{+}+Bq_\ell ^{-}||_\infty \nonumber \\= & {} \left\| \xi ^0_{p_1,\ell }\right\| _\infty ,\nonumber \\ || \xi _{p_{2,\ell }}||_\infty= & {} \left\| \bar{a}-\frac{1}{\sigma ^{+}} \bar{q}_\ell ^{+}-\frac{1}{\sigma ^{-}}\bar{q}_\ell ^{-} -\bar{x}_\ell \right\| _\infty \nonumber \\= & {} \left\| -\frac{1}{\sigma ^{+}}\bar{q}_\ell ^{+}-\frac{1}{\sigma ^{-}} \bar{q}_\ell ^{-}\right\| _\infty \nonumber \\\le & {} \frac{0.2\mu _0^{\frac{1}{2}}}{\sigma _{min}}, \end{aligned}$$

where $\sigma _{min} =\min \{\sigma ^{-},\sigma ^{+}\}$ and $|| \xi ^0_{p_{1,\ell }}||$ is the infeasibiliy from the prior problem. The expressions in (34) illustrate that primal infeasibility is expected to be small and therefore should not be an issue.

#### Dual infeasibility

The last three dual infeasibilties in (11) can be easily shown to be $(\xi _{d_{2,\ell }}, \xi _{d_{3,\ell }},\xi _{d_{4,\ell }})=(0,0,0)$ from (32) by direct substitution. However, for $\xi _{d_{1,\ell }}$, we have

35 $$\begin{aligned} \left\| \xi _{d_{1}}\right\| _\infty= & {} \left\| \bar{l}+\sum _\ell \bar{y}_\ell -\bar{s}_a\right\| _\infty \nonumber \\\le & {} \left\| 2\left| \left( \bar{l}-\sigma _{max}\sum _\ell \left| \bar{B}^Tu_\ell \right| \right) \right| +(2n_L+1)\mu _0^{\frac{1}{2}}e\right\| _\infty . \end{aligned}$$

This is the violation of the first dual constraint in (11) which is actually proportional to the magnitude of the dual violations used as criteria for adding the members, see (12). Such violation may be considerable, especially in the early member adding iterations. The warm starting routine [11, 13] will be applied to absorb it.

#### Centrality

In order to assess the centrality of the new point, we will compute complementarity products for all newly added variables. The pairs $(\bar{a},\bar{s}_a)$ are $\mu _0$-centered from (33). Below, we evaluate the remaining complementarity products $(\bar{q}_\ell ^{+},\bar{s}_{q_\ell ^{+}})$, $(\bar{q}_\ell ^{-},\bar{s}_{q_\ell ^{-}})$, $(\bar{x}_\ell ,\bar{s}_{x_\ell })$, $\ell \in \{1,\ldots ,n_L\}$.

36 $$\begin{aligned} (\bar{q}_\ell ^{+})_j\left( \bar{s}_{q_\ell ^{+}}\right) _j= & {} 0.1\mu _0^{\frac{1}{2}}\left( -\bar{B}^Tu_\ell -\frac{1}{\sigma ^{+}}\bar{y}_\ell \right) _j\nonumber \\= & {} 0.1 \mu _0^{\frac{1}{2}}\left( -\bar{B}^Tu_\ell +\frac{\sigma _{max}}{\sigma ^{+}} |\bar{B}^Tu_\ell |+\frac{1}{\sigma ^{+}}\mu _0^{\frac{1}{2}} e\right) _j, \end{aligned}$$

37 $$\begin{aligned} (\bar{q}_\ell ^{-})_j\left( \bar{s}_{q_\ell ^{-}}\right) _j= & {} 0.1\mu _0^{\frac{1}{2}}\left( \bar{B}^Tu_\ell -\frac{1}{\sigma ^{+}}\bar{y}_\ell \right) _j\nonumber \\= & {} 0.1\mu _0^{\frac{1}{2}} \left( \bar{B}^Tu_\ell +\frac{\sigma _{max}}{\sigma ^{-}}|\bar{B}^Tu_\ell | +\frac{1}{\sigma ^{-}}\mu _0^{\frac{1}{2}} e\right) _j. \end{aligned}$$

The above two equations show that for any $j\in K$, either of the products $(\bar{q}_\ell ^{+})_j(\bar{s}_{q_\ell ^{+}})_j$ or $(\bar{q}_\ell ^{-})_j(\bar{s}_{q_\ell ^{-}})_j$ is always nearly $\mu _0$-centered when $\sigma ^{+}=\sigma ^{-}$ depending on the sign of $(\bar{B}^Tu_\ell )_j$. Nevertheless, using the fact that the maximum number of non-zero entries in each column of $\bar{B}$ is 2*N* for *N*-dimensional problem and the absolute value of these entries does not exceed unity, we have

$$\begin{aligned} |(\bar{B}^Tu)_j|_\ell \le 2N|u_\ell |_\infty ,\;N\in \{2,3\}. \end{aligned}$$

Then, we get the following general estimate.

38 $$\begin{aligned} \frac{0.1}{\sigma ^{+}}\mu _0\le & {} (\bar{q}_\ell ^{+})_j \left( \bar{s}_{q_\ell ^{+}}\right) _j \le 0.1\mu _0^{\frac{1}{2}} \left( 2\frac{\sigma _{max}}{\sigma ^{+}}|\bar{B}^Tu_\ell | +\frac{1}{\sigma ^{+}}\mu _0^{\frac{1}{2}} e\right) _j\nonumber \\\le & {} \frac{0.1}{\sigma ^{+}}\mu _0+0.4\mu _0^{\frac{1}{2}} N\frac{\sigma _{max}}{\sigma ^{+}}|u_\ell |_\infty ,\;\forall j\in K. \end{aligned}$$

Similarly,

39 $$\begin{aligned} \frac{0.1}{\sigma ^{-}}\mu _0\le (\bar{q}_\ell ^{-})_j \left( \bar{s}_{q_\ell ^{-}}\right) _j \le \frac{0.1}{\sigma ^{-}}\mu _0 +0.4\mu _0^{\frac{1}{2}}N\frac{\sigma _{max}}{\sigma ^{-}}|u_\ell |_\infty ,\;\forall j\in K. \end{aligned}$$

Finally,

40 $$\begin{aligned} (\bar{x}_\ell )_j\left( \bar{s}_{x_\ell }\right) _j =-(\bar{a})_j(\bar{y}_\ell )_j= & {} -\mu _0\frac{(\bar{y}_\ell )_j}{(\bar{s}_a)_j}\nonumber \\= & {} -\mu _0\frac{(\bar{y}_\ell )_j}{\max \left\{ \left| \bar{l}_j +\sum _\ell (\bar{y}_\ell )_j\right| ,\mu _0^{\frac{1}{2}}\right\} }\nonumber \\\le & {} -\mu _0^{\frac{1}{2}}(\bar{y}_\ell )_j =\mu _0^{\frac{1}{2}}\left( \sigma _{max}|(\bar{B}^Tu_\ell )_j| +\mu _0^{\frac{1}{2}}\right) \nonumber \\\le & {} \mu _0+2\mu _0^{\frac{1}{2}} N|u_\ell |_\infty . \end{aligned}$$

On the other hand,

41 $$\begin{aligned} (\bar{x}_\ell )_j\left( \bar{s}_{x_\ell }\right) _j= & {} -\mu _0\frac{(\bar{y}_\ell )_j}{\max \left\{ \left| \bar{l}_j +\sum _\ell (\bar{y}_\ell )_j\right| ,\mu _0^{\frac{1}{2}}\right\} }\nonumber \\= & {} \mu _0\frac{\left( \sigma _{max}|(\bar{B}^Tu_\ell )_j| +\mu _0^{\frac{1}{2}}\right) }{\max \left\{ \left| \bar{l}_j-\sigma _{max} \sum _\ell \right| (\bar{B}^Tu_\ell )_j\left| -n_L\mu _0^{\frac{1}{2}}\right| , \mu _0^{\frac{1}{2}}\right\} } \nonumber \\\ge & {} \mu _0\frac{\left( \sigma _{max}|(\bar{B}^Tu_\ell )_j| +\mu _0^{\frac{1}{2}}\right) }{\max \left\{ \sigma _{max}\sum _\ell | (\bar{B}^Tu_\ell )_j|+n_L\mu _0^{\frac{1}{2}}, \mu _0^{\frac{1}{2}}\right\} } \nonumber \\= & {} \mu _0\frac{\left( \sigma _{max}|(\bar{B}^Tu_\ell )_j| +\mu _0^{\frac{1}{2}}\right) }{ \sigma _{max}\sum _\ell |(\bar{B}^Tu_\ell )_j| +n_L\mu _0^{\frac{1}{2}} }\nonumber \\\ge & {} \mu _0\frac{\mu _0^{\frac{1}{2}}}{2n_L\sigma _{max}N\max _{\ell } |u_\ell |_\infty +n_L\mu _0^{\frac{1}{2}} }\nonumber \\= & {} \mu _0 \frac{1}{ n_L\left( 2\sigma _{max}\mu _0^{\frac{-1}{2}} N \max _{\ell }|u_\ell |_\infty +1\right) } \end{aligned}$$

Then,

42 $$\begin{aligned} \frac{1}{n_L\left( 2\sigma _{max}\mu _0^{\frac{-1}{2}} N\max _{\ell }|u_\ell |_\infty +1\right) } \mu _0\le (\bar{x_\ell })_j(\bar{s}_{x_\ell })_j \le \mu _0+2\mu _0^{\frac{1}{2}} N|u_\ell |_\infty ,\;\forall j\in K. \end{aligned}$$

The estimates in (38), (39), and (42) show that the pairs $(\bar{q}_\ell ^{+},\bar{s}_{q_\ell ^{+}})$, $(\bar{q}_\ell ^{-},\bar{s}_{q_\ell ^{-}})$, $(\bar{x}_\ell ,\bar{s}_{x_\ell })$, $\ell \in \{1,\ldots ,n_L\}$ have no significant outliers from the $\mu _0$-centrality because the shift-like terms involving $|u_\ell |_\infty$ in the upper bounds are multiplied by $\mu _0^{\frac{1}{2}}$. Therefore, their contribution to the violation of $\mu _0$-centrality is reduced to some extent. Moreover, these terms are found out to be small in practice.

## Implementation, algorithmic parameters, and problem data

The interior point method is implemented in MATLAB (R2016a). All numerical experiments are performed on Intel(R) Core(TM) i5-4590T CPU, running at 2.00 GHz with 16 GB RAM. The interior point iterations stop when

43 $$\begin{aligned} \frac{||\xi ^k_p||_\infty }{1+||b||_\infty }\le \epsilon _p, \;\frac{||\xi ^k_d||_\infty }{1+||c||_\infty }\le \epsilon _d, \;\text {and}\;\frac{|c^Tx^k-b^Ty^k|}{1+|c^Tx^k|}\le \epsilon _{opt}, \end{aligned}$$

or $\mu <10^{-10}$, where $\xi _p$ and $\xi _d$ are given as (4).

### Algorithmic parameters

The initial points for the case of cold-start are set $x^0$, $s^0$ both to unity and $y^0$ to zero. The feasibility tolerances in (43) are set as $\epsilon _p=\epsilon _d=10^{-6}$ when the linear systems (17) are solved using direct methods. Otherwise $\epsilon _p=\epsilon _d=10^{-5}$. For the optimality tolerance, we use loose tolerances in the first few member adding iterations and then tighter in the last ones, i.e., $\epsilon _{opt}=[10^{-2},10^{-2},10^{-2},10^{-2},$ $10^{-3},10^{-4}]$, and then always $10^{-5}$, unless specified. We use $\tau =0.95$ in (7) for the step lengths and $\beta =0.001$ in (12) for the the member adding criteria.

### The preconditioner

**Fig. 4:**
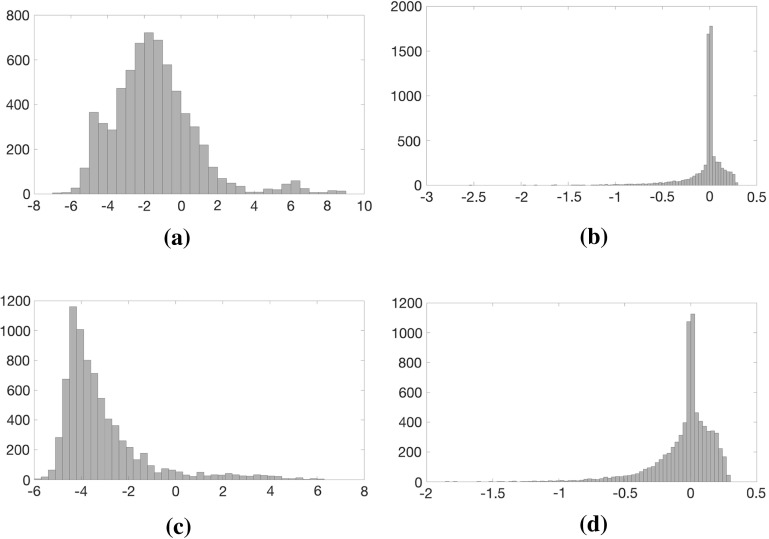
Eigenvalue distributions of the unpreconditioned $\tilde{B}\tilde{D}\tilde{B}^T$ and preconditioned $M^{-1}\tilde{B}\tilde{D}\tilde{B}^T$ matrices for the problems Example 5a I and Example 5c I in Table 1 for the final LPs and during the last interior point iterations. Logarithmic scale for eigenvalue magnitude is used. **a** Example 5a I: Eigenvalue distribution of $\tilde{B}\tilde{D}\tilde{B}^T$. **b** Example 5a I: Eigenvalue distribution of $M^{-1}\tilde{B}\tilde{D}\tilde{B}^T, \ \hbox {nnz} (M)/\hbox {nnz}(\tilde{B}\tilde{D}\tilde{B}^T)=0.2003$. **c** Example 5c I: Eigenvalue distribution of $\tilde{B}\tilde{D}\tilde{B}^T$. **d** Example 5c I: Eigenvalue distribution of $M^{-1}\tilde{B}\tilde{D}\tilde{B}^T, \ \hbox {nnz} (M)/\hbox {nnz}(\tilde{B}\tilde{D}\tilde{B}^T)=0.0915$

In case of the cold-start, we use the simple preconditioner (29) as long as $\mu >10^{-3}$ since there is a relative uniformity in the diagonal elements of the matrix $\tilde{D}$ in (18). However, when $\mu \le 10^{-3}$ or in general when the warm-starting strategy is invoked, we use the splitting preconditioner (26). To determine the threshold parameter $\delta$ in (23), recall that *m* is the number of non-fixed degrees of freedom and $n(\gg m)$ is the number of bars. We then set $\delta$ to be the smallest of the largest *m* diagonal elements in $\tilde{D}$ in (18) for the two-dimensional problems, and the smallest of the largest $\left[ m/3\right]$ elements for the three-dimensional problems. This choice is based on performing many numerical experiments and at this stage we do not have any theoretical justification why we needed a larger number of the diagonal elements in $\tilde{D}$ for two-dimensional problems compared to that of the three-dimensional problems. We have included Fig. 4 to give an insight into the eigenvalue distribution of the unpreconditioned $\tilde{B}\tilde{D}\tilde{B}^T$and preconditioned $M^{-1}\tilde{B}\tilde{D}\tilde{B}^T$ matrices for small size two- and three-dimensional problems Example 5a I and Example 5c I in Table 1. The distributions are shown for the final linear programs in the member adding scheme and the last interior point iteration. The histograms display the number of eigenvalues of a given magnitude. The Fig. 4a and c show that eigenvalues of the upreconditioned matrices are spread over a large range roughly between $10^{-7}{-}10^{8}$ for the two-dimensional and $10^{-6}{-}10^{6}$ for the three-dimensional problems. However, for the preconditioned matrices the distribution clusters within the interval roughly 0.003–2 for the two-dimensional problem and 0.01–2 for the three-dimensional problem, see Fig. 4b and d. Moreover, most of these eigenvalues are clustered near 1.

**Table 1:** Problem statistics for the problems in Fig. 5 for three level of nodal density distributions

Problems	Nodal densities	Number of potential bars	Number of DOF	Load cases
Example 5a I	$41\times 81$	5,512,860	16,538,580	1
Example 5a II	$61\times 121$	27,235,890	81,707,670	1
Example 5a III	$81\times 161$	85,027,320	255,081,960	1
Example 5b I	$41\times 81$	5,512,860	27,564,300	2
Example 5b II	$61\times 121$	27,235,890	136,179,450	2
Example 5b III	$81\times 161$	85,027,320	425,136,600	2
Example 5c I	$21\times 11\times 11$	3,227,070	9,681,210	1
Example 5c II	$29\times 15\times 15$	21,284,550	63,853,650	1
Example 5c III	$41\times 21\times 21$	163,452,240	490,356,720	1
Example 5d I	$9\times 9\times 25$	2,049,300	10,246,500	2
Example 5d II	$13\times 13\times 37$	19,546,878	97,734,390	2
Example 5d III	$17\times 17\times 49$	100,259,880	501,299,400	2

The number of degrees of freedom (DOF) is based on the problem formulation (10), i.e, for the variables $(a,q_\ell ^{+},q_\ell ^{-}).$ See also Remark 7

For the preconditioned conjugate gradient method (pcg), we set the maximum number of iterations to 100 when the simple preconditioner (29) is used and to 80 when the splitting preconditioner (26) is used. The tolerance is set to $\max \{\min \{10^{-4},0.1||\xi ||_2\},10^{-6}\}$, where $\xi$ is the residual given as in (4), i.e, we use tighter tolerance for the pcg when iterates are close to optimality.

#### Remark 5

For all of the two-dimensional problems in our numerical experiments, we always use direct methods for solving the linear systems (17) unless $n> 4m$ including the case when the option is set to use iterative methods. This is because, direct methods significantly outperform the iterative ones as long as *n* remains comparable to *m*. In practice, we observed that this happens only in the first two member adding iterations for the AP3 filtering approach which is our choice.

### The warm-start

If the option to use the warm-starting strategy is set to ’on’, then it is activated as early as in the third member adding iteration. We use the solutions obtained with tolerance $\varepsilon _{opt}=0.1$ for warm-starting the third and fourth, $\varepsilon _{opt}=0.01$ for the fifth and sixth, and $\varepsilon _{opt}=0.001$ for the seventh and the other subsequent problem instances in the member adding iterations. This is because, the similarity between the problems increases as we approach the last stages of the member adding iterations.

#### Remark 6

When the problems are too close, i.e., when only very few members are added the initial points used in warm-starts often correspond to optimality tolerances which are smaller than the prescribed ones 0.001–0.1. In that case, we save the solution obtained after the third interior point iteration.

### Problem data

**Fig. 5:**
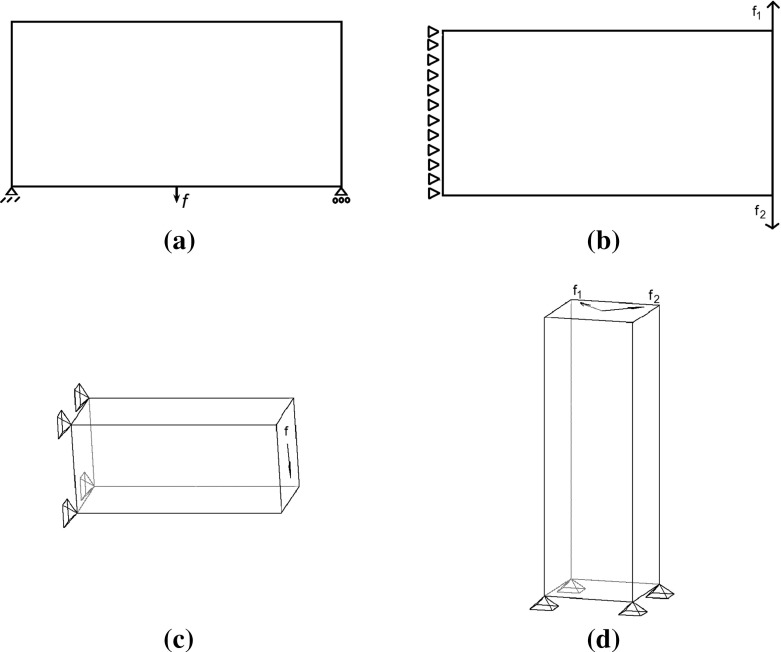
Design domains, boundary conditions, and loads

**Fig. 6:**
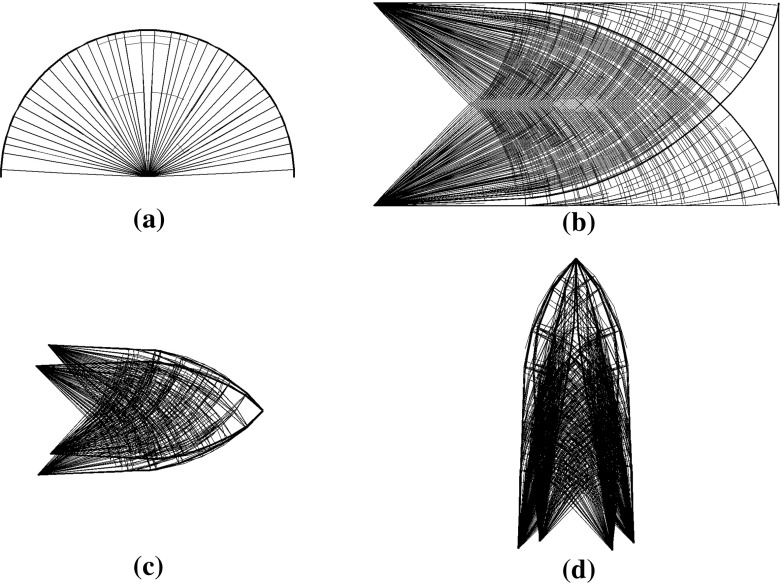
Optimal designs for the problems in Fig. 5. The bars shown are those with cross-sectional area $\ge 0.001a_{\max }$

For the numerical experiments, we solve the problems displayed in Fig. 5. The loads are all nodal of unit magnitude. The dimensions of the design domains are $1\times 2$, $1\times 2$, $2\times 1\times 1$, and $1\times 1\times 3$ of unit lengths. The problems are solved for three levels of nodal density distributions. The problem instances are named as Example 5a I, Example 5a II, Example 5a III, Example 5b I and so on. Their statistics are given in Table 1. The number of degrees of freedom is based on the problem formulation (10), i.e, for the variables $(a,q_\ell ^{+},q_\ell ^{-}).$ See also Remark 7. In all cases, we consider equal tensile and compressive strengths of unity. The optimal designs are given in Fig. 6 where the bars shown are those with cross-sectional area $\ge 0.001a_{\max }$.

#### Remark 7

Note that the size of the corresponding standard linear program of the form (1) with the matrix *A* in (14) can be obtained from Table 1. For example, for the three-dimensional problem Example 5c III with 163, 452, 240 bars, the corresponding standard linear program (1) will have 653, 808, 960 primal variables and 163, 506, 471 constraints. Of course, by applying the member adding technique we avoid the need of dealing with problems of such excessive sizes.

**Table 2:** Numerical results comparing AP1, AP2, and AP3 for the problem Example 5a I in Table 1

	AP1	AP2			AP3	All bars
		$\alpha = 0.05$	$\alpha =0.1$	$\alpha = 1$		
Volume	3.14725	3.14719	3.14722	3.14718	3.14720	3.14753
Final num. of bars	1,087,898	32,394	34,514	78,382	41,952	5,512,860
Mem. add. iter.	3	34	21	9	12	1
Total CPU (s)	558	121	70	50	25	1663

The linear systems (17) are solved using direct methods. Warm-start strategy activated at the third member adding iterations. For AP1, $\epsilon _{opt}=[10^{-2},10^{-4},10^{-5},\ldots ]$ and when all bars are used as the last column $\epsilon _{opt}=10^{-5}$

**Table 3:** Numerical results comparing AP2 and AP3 for the problems Example 5a II and Example 5a III in Table 1

	Example 5a II				Example 5a III			
	AP2			AP3				AP3
	$\alpha =0.05$	$\alpha =0.1$	$\alpha =1$		$\alpha =0.05$	$\alpha =0.1$	$\alpha =1$	
Volume	3.14508	3.14505	3.14502	3.14509	3.14399	3.14396	3.14393	3.14396
Final num. bars	77,497	90,826	221,822	100,508	141,657	167,446	463,324	197,132
Mem add. iter.	40	26	12	9	42	30	13	11
Total CPU (s)	544	356	385	90	1367	1087	1269	282

The linear systems (17) are solved using direct methods. Warm-start strategy activated at the third member adding iterations

**Table 4:** Numerical results comparing the overall performance of the cold-start and warm-start strategies for some of the problems in Table 1

Problems	Cold-start					Warm-start				
	Final num. of bars	Mem add. iter.	IPM iter. (final LP)	CPU (s)	Volume	Final num. of bars	Mem add. iter.	IPM iter. (final LP)	CPU (s)	Volume
Example 5a I	42,036	11	30	67	3.14723	41,952	12	3	25	3.14720
Example 5a II	102,222	9	38	188	3.14506	100,508	9	10	90	3.14509
Example 5b I	46,246	10	31	300	7.79650	48,320	8	14	110	7.79648
Example 5b II	115,324	9	35	960	7.79149	120,616	9	7	448	7.79150
Example 5c I	44,810	7	26	122	7.46812	45,082	6	10	70	7.46812
Example 5c II	142,884	9	31	1396	7.42692	143,188	8	6	621	7.42743
Example 5d I	43,552	7	28	384	19.99714	44,936	7	12	257	19.99714
Example 5d II	193,764	10	37	38,916	19.85804	202,716	8	9	10687	19.85839

The linear systems (17) are solved using direct methods. AP3 filtering approach is used

**Table 5:** Comparison of the number of interior point iterations when using cold-start and warm-start strategies

Mem add. iter.		1	2	3	4	5	6	7	8	9	10	11	12
Example 5a I	IPM iter. cold-start	10	13	18	19	23	28	30	31	31	31	30	–
	IPM iter. warm-start	10	13	13	11	11	11	10	12	10	6	5	3
Example 5a II	IPM iter. cold-start	11	14	20	22	26	32	37	37	38	–	–	–
	IPM iter. warm-start	11	14	13	13	13	18	15	13	10	–	–	–
Example 5b I	IPM iter. cold-start	13	17	17	19	23	28	30	30	30	31	–	–
	IPM iter. warm-start	13	17	17	12	12	13	18	14	–	–	–	–
Example 5b II	IPM iter. cold-start	15	20	20	22	28	32	36	35	35	–	–	–
	IPM iter. warm-start	15	20	21	15	16	13	16	10	7	–	–	–
Example 5c I	IPM iter. cold-start	12	16	18	17	21	23	26	–	–	–	–	–
	IPM iter. warm-start	12	16	20	15	14	10	–	–	–	–	–	–
Example 5c II	IPM iter. cold-start	14	20	22	22	26	39	32	32	31	–	–	–
	IPM iter. warm-start	14	20	24	17	14	11	9	6	–	–	–	–
Example 5d I	IPM iter. cold-start	14	17	19	19	22	26	28	–	–	–	–	–
	IPM iter. warm-start	14	17	19	15	13	15	12	–	–	–	–	–
Example 5d II	IPM iter. cold-start	16	21	24	23	28	33	40	37	37	37	–	–
	IPM iter. warm-start	16	21	21	19	16	13	12	9	–	–	–	–

The linear systems (17) are solved using direct methods. AP3 filtering approach is used

**Table 6:** Numerical results comparing the use of direct and iterative methods for solving the linear systems (17) for some of the problems listed in Table 1

Problems	Direct methods				Iterative methods						
	Final num. bars	Mem add. iter.	CPU (s)	Volume	Final num. bars	Mem add. iter	CPU (s)	Volume	Final LP		
									IPM iter.	Pcg iter.	
										Predictor	Corrector
Example 5a I	41,952	12	25	3.14720	42,093	9	24	3.14726	10	190	249
Example 5a II	100,508	9	90	3.14509	104,145	9	79	3.14512	9	196	388
Example 5b I	48,320	8	110	7.79648	47,658	8	71	7.79659	12	211	190
Example 5b II	120,616	9	448	7.79150	118,672	10	400	7.79153	15	412	541
Example 5c I	45,082	6	70	7.46812	45,004	6	15	7.46812	10	73	67
Example 5c II	143,188	8	621	7.42743	143,567	8	77	7.42741	6	30	71
Example 5d I	44,936	7	257	19.99714	45,013	7	56	19.99725	14	116	110
Example 5d II	202,716	8	10687	19.85839	195,630	9	381	19.85815	7	37	81

Warm-start strategy activated at the third member adding iteration and AP3 filtering approach used

**Table 7:** Numerical results for using MOSEK and the interior point method of this paper for problems listed in Table 1

Problems	Mosek				IPM of this paper			
	Final num. bars	Mem add. iter.	CPU (s)	Volume	Final num. bars	Mem add. iter.	CPU (s)	Volume
Example 5a I	43,266	9	24	3.14722	42,093	9	24	3.14726
Example 5a II	107,266	12	135	3.14506	104,145	9	79	3.14512
Example 5a III	202,208	9	233	3.14395	200,967	8	215	3.14399
Example 5b I	47,148	8	117	7.79645	47,658	8	71	7.79659
Example 5b II	120,979	8	451	7.79140	118,672	10	400	7.79153
Example 5b III	233,658	8	1126	7.78946	220,136	9	796	7.78946
Example 5c I	50,054	13	89	7.46811	45,004	6	15	7.46812
Example 5c II	144,053	8	304	7.42740	143,567	8	77	7.42741
Example 5c III	495,318	10	3175	7.40381	495,013	12	738	7.40383
Example 5d I	46,196	8	250	19.99713	45,013	7	56	19.99725
Example 5d II	211,060	12	5246	19.85793	195,630	9	381	19.85815
Example 5d III	718,067	12	58,137	19.79508	694,505	12	2527	19.79499

For the IPM of this paper, the linear systems (17) are solved using iterative methods except during the first two member adding iterations for the two-dimensional problems. Warm-start strategy is activated at the third member adding iteration and AP3 filtering approach is used

### Starting structures

We always start with the structures shown in Fig. 1a for two-dimensional problems and Fig. 1b for three-dimensional problems and then upgrade these structures based on the member adding procedure described in Sect. 3.1.

## Numerical results

The reported CPU times correspond only to solving the optimization problems. In Tables 2, 3, 4, 5, 6 and 7, the label “Final num. of bars” refers to the number of bars considered in the linear programming problem formulation of the last member adding iteration and not the number of bars with non-zero cross-sectional area.

### The filtering approaches

We compare the three filtering approaches AP1, AP2, and AP3 in the member adding process described in Sect. 3.1 applied to test problem Example 5a. It is solved for three nodal densities

using the interior point method described in this paper where the linear systems (17) are solved using direct methods, and the warm-start strategy is used. The numerical results are presented in Tables 2 and 3. In the first table, we present all approaches that additionally include the case when the problem is solved for all potential member bars. In the second table, we compare only AP2 and AP3 as the problem is larger in this case. In general, the results illustrate two outcomes. The first one is the efficiency of the member adding method, i.e, it obtains solutions using approximately $1\%$ of all the possible members and it needs significantly less time than a method which uses all potential bars, see columns 6 and 7 in Table 2. The second observation is that the approach AP3 seems to outperform the others. It obtains solutions faster by using fewer member adding iterations keeping the sizes of the problems small enough, somewhere between those obtained by strategy AP2 with $\alpha =0.1$ and $\alpha =1$. We have also noticed this behaviour has been consistent for the other examples. Based on these results, AP3 becomes our method of choice and we follow this approach in all of the next examples.

### Cold-start versus warm-start

In Table 4, we present numerical results to compare the warm-start and cold-start strategies. The linear systems (17) are solved using direct methods. As it can be seen in columns 5 and 10, the use of the warm-start strategy reduces the computational time. This is achieved by reducing the number of interior point iterations, see Table 5.

#### Remark 8

**Fig. 7:**
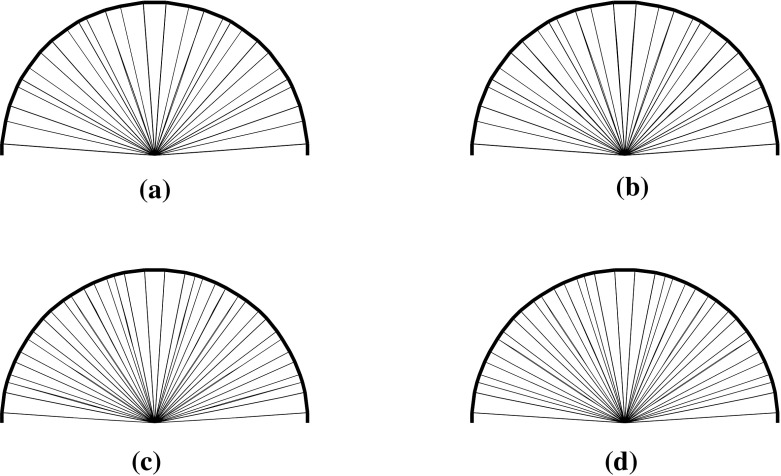
Optimal designs for the problems Example 5a I and Example 5a II for cold-start and warm-start strategies. The bars shown are those with cross-sectional area $\ge 0.001a_{\max }$. **a** Example 5a I, cold-start. **b** Example 5a I, warm-start. **c** Example 5a II, cold-start. **d** Example 5a II, warm-start

In the early member adding iterations, warm-starting strategy is able to save merely a few interior point iterations. However, in later stages of the member adding, when only a few new members are added and the problem instances do not change significantly, the warm-starting technique is very successful. In Table 5 the full history of the interior point iterations is reported for a number of examples with cold-start and warm-start strategies used. Additionally, in Fig. 7 we present the corresponding optimal designs obtained with cold start and warm start strategies, respectively. The reader will observe that there is no noticeable difference in the optimal solutions obtained with these two variants of the starting strategies.

#### Remark 9

We have observed that, the efficiency of the warm-start strategy further improves when the iterative methods are used to solve the linear systems. This is because it promotes the early start of the more efficient splitting preconditioner.

### Direct methods versus iterative methods

In this section, we present numerical results to make comparisons between the use of the direct and iterative methods to solve linear system (17). For direct methods, we use the Matlab built-in function chol to find the Cholesky factorization of the coefficient matrix. Once again, we consider some of the problems listed in Table 1 and report their solution statistics in Table 6. The computational times reported in columns 4 and 8 demonstrate the efficiency of the iterative methods specially for the three-dimensional problems, see the CPU times for Example 5d II in Table 6. There are two main reasons why their efficiency for two-dimensional problems is not as pronounced as for three-dimensional ones. Firstly, as pointed out in Sect. 7, we needed more non-zero entries of the diagonal matrix $\tilde{D}$ to determine the splitting preconditioner for the two-dimensional problems. This makes the preconditioner more dense and more expensive. Secondly, the linear systems solved to compute the predictor and corrector directions require relatively larger number of pcg iterations for two-dimensional problems, see columns 11 and 12 of Table 6. These pcg iterations seem to be reduced if we consider a denser preconditioner, but this leads to longer run times.

It is worth mentioning that three-dimensional problems are more relevant for practical applications. However, they involve more complicated grids and this causes a quick loss of sparsity in computations. The use of iterative linear algebra solver produces more spectacular savings over direct methods in this case.

### Large-scale problems

In this section, we consider solutions of all the problem instances listed in Table 1 including the ones with the finest nodal density distributions. We include numerical results when the problems are solved by a commercial solver MOSEK (version 7) [27] with Matlab interface. This is to give an insight into the overall performance of the interior point method of this paper and the techniques employed in reference to existing solvers. For MOSEK, we set the algorithm to interior point method, the maximum number of interior point iterations to 70, pre-solve ’on’, the primal and dual feasibility tolerances $10^{-6}$, a member adding dependant optimality tolerance $[10^{-2},10^{-2},10^{-2},10^{-2},$ $10^{-3},10^{-4}]$, and then always $10^{-5}$. This is similar to the settings presented in Sect. 7 for our implementation.

#### Remark 10

The numerical results in Table 7 for using MOSEK and our version of interior point method should not be considered as a direct comparison between the two solvers for many reasons. There are important differences between these two solvers. First, the implementations use different programming languages. Moreover, MOSEK uses direct methods to solve linear systems in its interior point implementation. In addition, the linear systems are reduced only to the normal equation systems (9) (or (15) for the truss layout problems of this paper) and not further to the smaller system (17). MOSEK uses powerful presolving which may significantly reduce the problem size and as a result simplify the normal equations. For example, MOSEK’s CPU with pre-solve turned ‘off’ for the first eleven problems in Table 7 is 75, 250, 522, 214, 605, 1890, 99, 785, 8413, 221, and 9555 s. Our solver does not use pre-solve. Finally, MOSEK uses its own default initial point and does not use a warm-start point.

The numerical results presented in Table 7, particularly columns 4 and 8, show that the IPM of this paper is competitive for two-dimensional problems and very efficient for three-dimensional problems, see the last four rows of these columns. Once again as mentioned in the discussion on direct versus iterative methods, most practical applications are spacial, i.e., three-dimensional problems, and they are more challenging to solve due to their excessive size. Therefore, having an efficient tool such as the one presented in this paper able to solve three-dimensional problems is paramount.

#### Remark 11

Note that for a very dense nodal distribution, the solution of test problem Example 5a is expected to be $\pi$ [10], and those of problems Example 5b and d are expected to converge to 7.78480 and 19.67476 which are the corresponding analytic solutions to the exact least-weight truss layout problems as shown in [31] and [34], respectively.

## Conclusions

We have described a primal-dual interior point method that exploits the algebraic structure of multiple-load plastic layout optimization of truss structures. The method is supported with a warm-start strategy that benefits from the closeness of the problems after performing few member adding iterations. Moreover, large linear systems arising in the interior point method are solved using Krylov type iterative method. The numerical results in Sect. 8 illustrate the overall efficiency of the method to solve large-scale problems. The method excels on three-dimensional problems which are more challenging due to high connectivity of the grids involved, and are significantly more important for practical engineering applications than two-dimensional problems.
